# Congenital cystic adenomatoid malformation – dangers of misdiagnosis: a case report

**DOI:** 10.1186/s13256-017-1349-5

**Published:** 2017-08-04

**Authors:** Wafae El Amraoui, Aziza Bentalha, Hajar Hamri, Salma Es-Chrif El Kettani, Alae El Koraichi

**Affiliations:** 1 Faculty of Medicine, University Mohammed 5, Rabat, Morocco; 2 Department of Anesthesiology, Hopital d’enfants de Rabat, Rabat, Morocco; 3 Department of Radiology, Hopital d’enfants de Rabat, Rabat, Morocco

**Keywords:** Congenital cystic adenomatoid malformation, Children, Clinical symptoms, Diagnosis, Treatment

## Abstract

**Background:**

Congenital cystic adenomatoid malformation is a rare pulmonary malformation, but is the most common lung malformation observed in children. In developing countries, such as Morocco, prenatal diagnosis is missing. Congenital cystic adenomatoid malformation may occur after birth in the presence of complications and needs a computed tomography scan for confirmation. However, our lack of awareness of this malformation has been responsible for a late and wrong diagnosis along with therapeutic errors. We report the first case in Morocco where diagnosis is confirmed by histology after death.

**Case presentation:**

A 10-month-old Arab boy was prescribed various antibiotics (including anti-staphylococcal) and endured repeated chest drainages, leading to his death just after radiological diagnosis and instant surgery.

**Conclusions:**

The goal of this case report is to firmly express the need for both pediatricians and radiologists to enlarge diagnosis investigations, especially of congenital or constitutional entities in children, as soon as recurrence of respiratory distress and pulmonary infections are manifested. We also emphasize this important entity because of its frequency, to avoid the eventual therapeutic errors.

## Background

Congenital cystic adenomatoid malformation (CCAM) is a rare malformation, with unknown causes, that affects distal bronchi [1]. It accounts for 25% of congenital pulmonary malformations and most cases are found in neonates and babies. It yields single or multiple cysts in different locations, with ipsilateral or even mediastinal pulmonary compression. The clinical manifestations are respiratory distress and recurrent pulmonary infection. CCAM is often misdiagnosed by radiology as a pulmonary cyst, bubbles of emphysema, or pneumothorax [1]. Our case report describes a late diagnosis of CCAM in a 10-month-old boy who was prescribed many antibiotics and who endured several lung drainages before being admitted to an operating room for a lobectomy.

We aim through this paper, to better understand this disease and its management and raise awareness about its seriousness. The rarity of this malformation, the difficulty of the diagnosis, and the quandary of the decision for an urgent elective surgical excision of lung, makes CCAM a real challenge for a multidisciplinary team including pediatricians, pediatric surgeons, pediatric intensivists, and radiologists.

## Case presentation

**Fig. 1:**
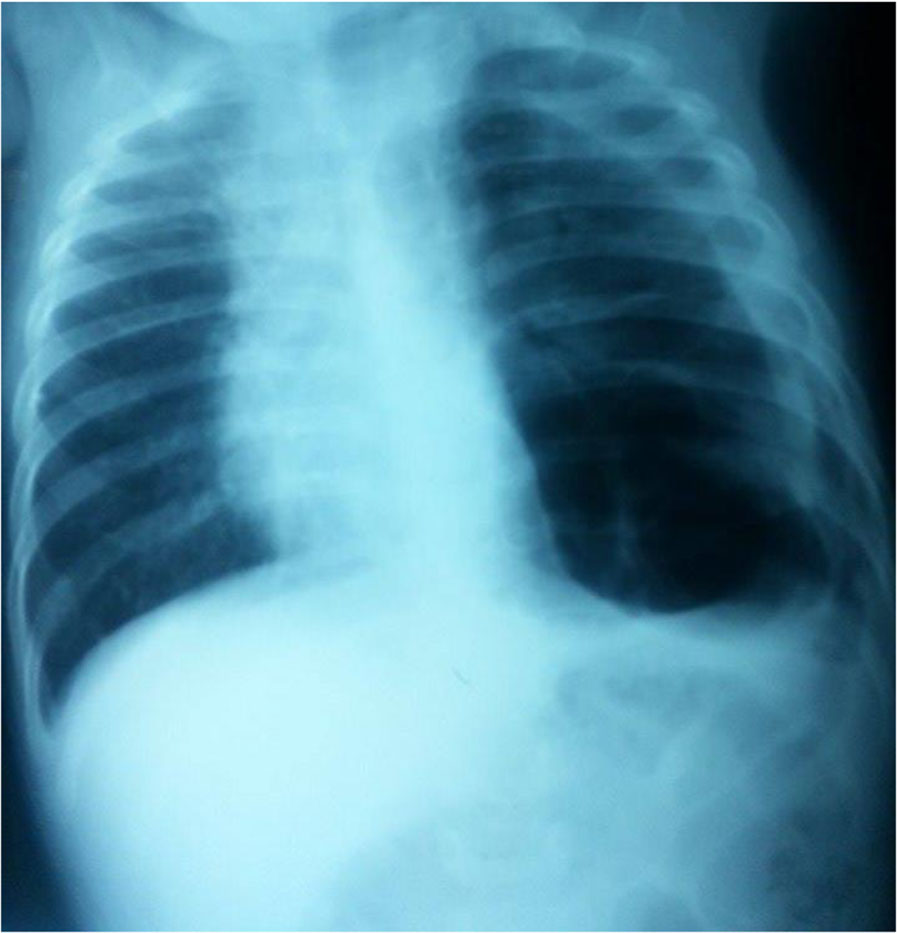
Thorax radiography showing pneumothorax

**Fig. 2:**
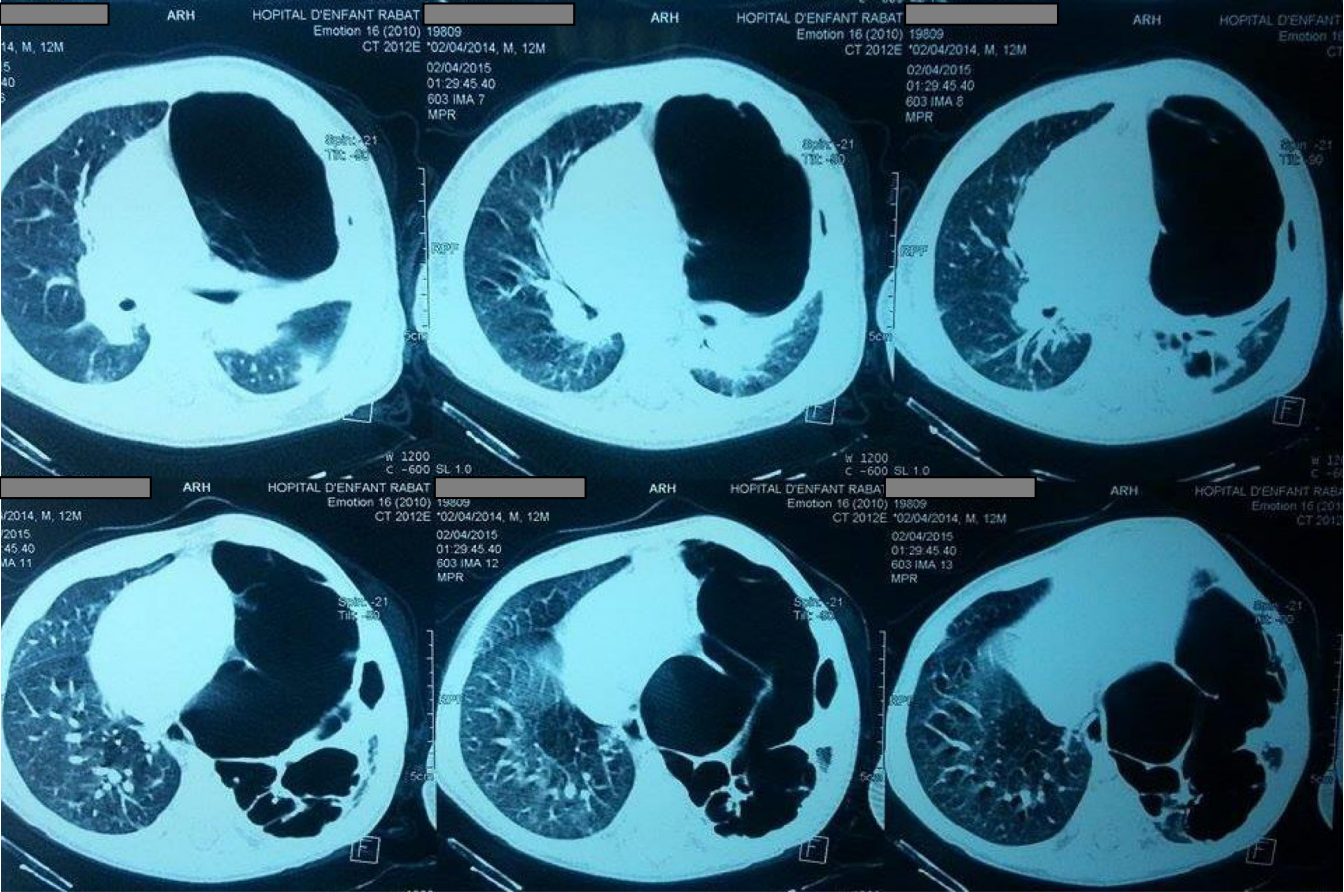
Scannographic image showing a compressive pneumothorax with huge bubbles of emphysema

A 10-month-old Arab boy born via a normal pregnancy without complications was admitted to intensive care for respiratory distress. Since birth, he had had several episodes of respiratory distress and pulmonary infections, which were successfully treated with antibiotics. He was diagnosed as having pleuropulmonary staphylococcal infection and was admitted to the pediatric service for 20 days. In fact, a computed tomography (CT) scan confirmed a localized pneumothorax suspected on radiography (Fig. 1) with multiple emphysema bubbles (Fig. 2). Thoracic drainage isolated a methicillin-susceptible *staphylococcus* on the collection of pus, and antibiotics were used with clinical improvement: intravenously administered ceftriaxone 100 mg/kg per day for 21 days, then orally relayed for another 3 weeks, initially associated to intravenously administered gentamycine 5 mg/kg per day for 5 days. He was discharged and an appointment was fixed within 10 days.

**Fig. 3:**
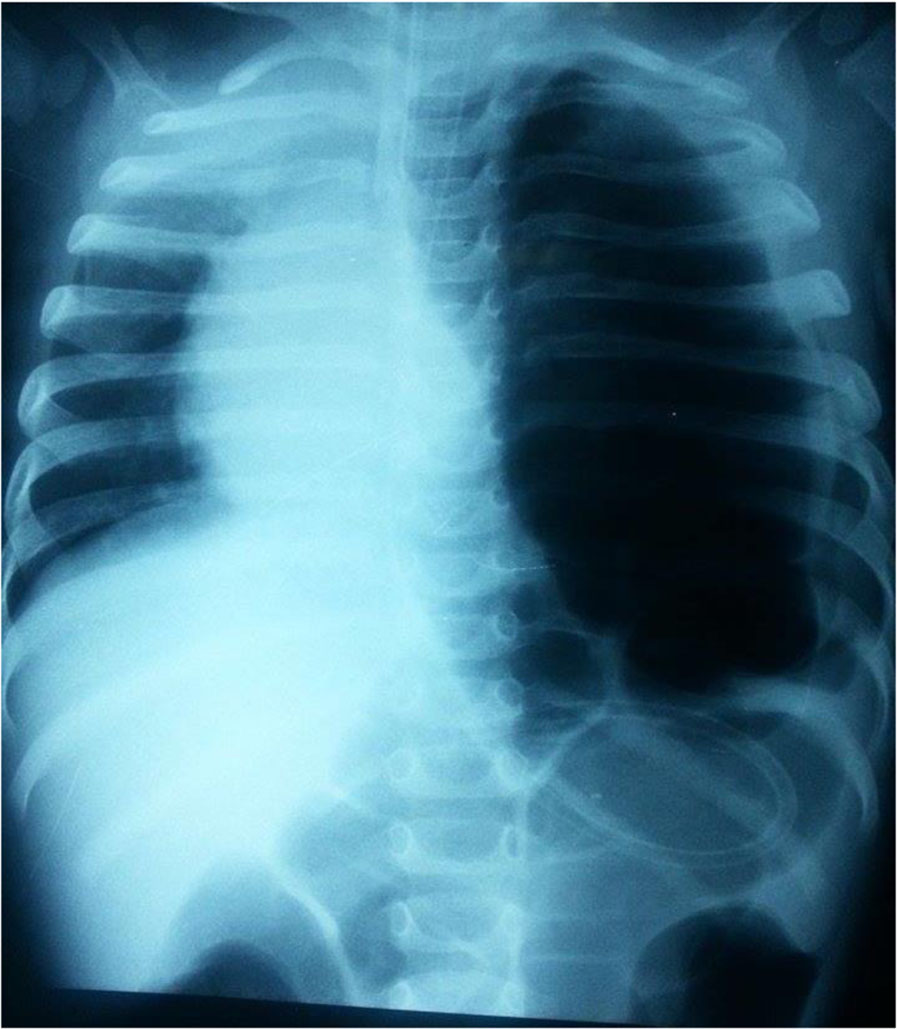
Thorax radiography suspecting a pneumothorax or a diaphragmatic hernia

A week later, at the current episode, he had fever and respiratory distress. Unable to distinguish between a pneumothorax and a diaphragmatic hernia (Fig. 3), we opted for a new CT scan. Pneumothorax was confirmed and re-drained immediately.

The re-reading of scanner plates, by an experienced radiologist with the help of pediatric surgeons, evoked the diagnosis of CCAM.

Symptoms were complicated a few hours later, with: hemodynamic instability; severe hypoxia, partial pressure of oxygen (pO_2_) 41 mmHg; hypercapnia, partial pressure of carbon dioxide (pCO_2_) 105 mmHg; and acidosis, pH 6.9.

After resuscitation measures, our patient was quickly transferred to the operating room for lobectomy of the lower lobe of his left lung. He died a few hours later due to hypoxia and hemodynamic instability refractory to resuscitation.

The pathological findings 10 days later of the surgical specimen confirmed the existence of CCAM.

**Fig. 4:**
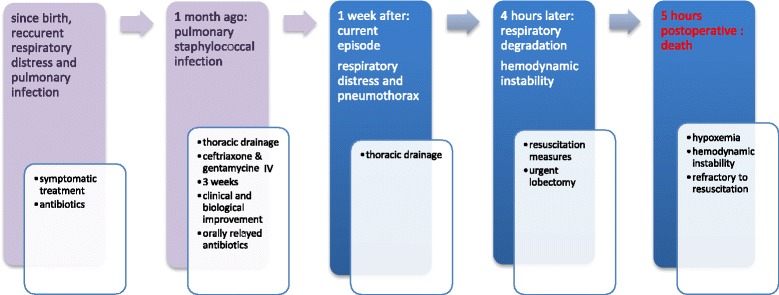
Timeline. *IV* Intravenous

The timeline of our patient is shown in Fig. 4.

## Discussion

CCAM or cystic lung malformation (CLM) is a rare congenital anomaly. However, it remains the most common malformation of lung development [2] (25% [3]). Its incidence is approximately 1 in 10,000 to 35,000 pregnancies, and its prevalence is approximately 9/100,000 births [4].

The etiology of CCAM is unknown. We assume a transient and focal anomaly of lung development, probably secondary to airway obstruction [2].

We have recently described maladjustment in pseudoglandular phase of lung development, before 16 weeks of gestation. This might be caused by the ceasing of bronchial maturation along with overgrowth of mesenchymal elements [4]. This yields multiple cysts at the terminal bronchioles with various sizes and variable locations [1, 5].

Currently, thanks to advances in prenatal imaging, pulmonary tract defects can be detected during pregnancy or at birth [6]. They may be responsible for hydramnios or hydrops *in utero*. In addition, prenatal ultrasound monitoring allows for perinatal care planning [1]. This is rarely the case in underdeveloped countries, where all pregnancies are not well attended, but also related to a deficit in experienced practitioners.

In postnatal conditions, CCAM are responsible for a large clinical and radiological polymorphism. In general they are asymptomatic; they can be complicated and manifest as fever, cough, or respiratory distress related to emphysema, mediastinal hernia, pulmonary hypoplasia, or respiratory infection [4]. These symptoms occur before the age of 2 in 80 to 85% of cases [2]. They are rarely discovered by spontaneous pneumothorax, particularly in adulthood [2, 6, 7]. All these conditions were found in our patient.

The imagery is based primarily on CT scan, but the diagnosis remains difficult because of its scarcity [4, 8]. It is necessary to resort to experienced radiologists to distinguish between CCAM and other pulmonary diseases (that is, pulmonary abscess, diaphragmatic hernia, and lung tumors) [3, 4]. In our case, the diagnosis could have been earlier if the first reading of the scanner was performed by an experienced radiologist. A misdiagnosis can even impose on children a full antitubercular treatment or, such as our case, repeated intercostal drainages [9].

**Fig. 5:**
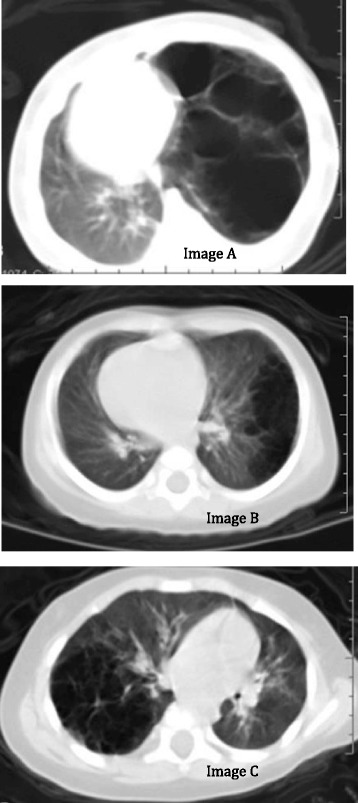
image **a**: type I of CCAM, large cystic lesion (diameter >2 cm) with a thick wall. image **b**: type II, Several cystic lesions (diameter < 1 cm) with numerous cavities. image **c**: type III, a solid mass encountered with tiny vesicles, with a significant mediastinal displacement

On radiological examination (Fig. 5), a CCAM can be classified into three types, corresponding to Stocker histopathological classification [1]:

- The most characteristic lesion for type I is the existence of a large cavity with a thick wall; diameter >2 cm (image A).
- Numerous cystic lesions with separate cavities characterize type II, the maximum diameter often being less than 1 cm (image B).
- For type III, which is extremely rare, a large solid mass is encountered with innumerable vesicles the size of an alveolus, exerting a significant mediastinal displacement (image C).

Magnetic resonance imaging or angiography may be necessary for a better delineation of vascularization in cases of pulmonary retraction [9]. We regret that our patient did not benefit from further investigations.

The clinical diagnosis, highly oriented by radiological approach, is confirmed by pathological examination [2].

Apart from the mentioned complications, the big risk of CCAM is to develop a bronchioloalveolar carcinoma or other type of malignant transformation (for example, sarcoma, or blastoma [9]).

However, nearly half of CCAM regresses after birth [10]; therefore, the decision for surgical excision in asymptomatic forms and its timing remain controversial [7, 9–12]. Some authors advocate clinical and radiological follow-up [1, 10], especially with regard to small malformations (≤3 cm [3]). The elective and early surgical excision of CCAM would be justified to avoid complications and malignant transformation [1.3]. It was proposed from the age of 3 to 6 months [8, 13].

Complicated forms are therapeutic emergencies: drainage *in utero* in macrocystic lesions, maternal corticosteroid in compressive microcystic forms, percutaneous sclerotherapy intrauterine, and early postnatal surgical resection [1, 5, 8]. To further reduce the risk of recurrence, some authors recommend not only the removal of the defect, but rather a lobectomy or pneumonectomy [11, 13]. In our case, lobectomy was carried out due to the seriousness of symptoms and their recurrence.

The evolution of CCAM surgery is usually favorable. It leads to a low rate of postoperative morbidity and mortality, shorter hospital stay, and mainly lowers the risk of recurrence [1, 3, 9, 13]. It can lead to more or less severe respiratory failure [8], such as in our case.

## Conclusions

While timely CCAM treatment gives good results, prenatally undiagnosed symptomatic lesions are hard to detect postnatally. A real awareness of this rare entity among pediatricians and radiologists should allow early diagnosis and proper treatment, avoiding the use of antibiotics, antituberculosis drugs, and chest drainage, which can be dangerous.
